# Region-Enhancing Network for Semantic Segmentation of Remote-Sensing Imagery

**DOI:** 10.3390/s21217316

**Published:** 2021-11-03

**Authors:** Bo Zhong, Jiang Du, Minghao Liu, Aixia Yang, Junjun Wu

**Affiliations:** 1 College of Computer Science and Technology, University of Posts and Telecommunications, Chongqing 400065, China; zhongbo@radi.ac.cn (B.Z.); S190231220@stu.cqupt.edu.cn (J.D.); 2 State Key Laboratory of Remote Sensing Science, Aerospace Information Research Institute, Chinese Academy of Sciences, Beijing 100101, China; yangax@radi.ac.cn (A.Y.); Wujj@radi.ac.cn (J.W.)

**Keywords:** semantic segmentation, remote sensing imagery (HRRSI), deep convolutional neural network, regional integrity of images

## Abstract

Semantic segmentation for high-resolution remote-sensing imagery (HRRSI) has become increasingly popular in machine vision in recent years. Most of the state-of-the-art methods for semantic segmentation of HRRSI usually emphasize the strong learning ability of deep convolutional neural network to model the contextual relationship in the image, which takes too much consideration on every pixel in images and subsequently causes the problem of overlearning. Annotation errors and easily confused features can also lead to the confusion problem while using the pixel-based methods. Therefore, we propose a new semantic segmentation network—the region-enhancing network (RE-Net)—to emphasize the regional information instead of pixels to solve the above problems. RE-Net introduces the regional information into the base network, to enhance the regional integrity of images and thus reduce misclassification. Specifically, the regional context learning procedure (RCLP) can learn the context relationship from the perspective of regions. The region correcting procedure (RCP) uses the pixel aggregation feature to recalibrate the pixel features in each region. In addition, another simple intra-network multi-scale attention module is introduced to select features at different scales by the size of the region. A large number of comparative experiments on four different public datasets demonstrate that the proposed RE-Net performs better than most of the state-of-the-art ones.

## 1. Introduction

Semantic segmentation of HRRSI, which classifies every pixel in an image, has been one of the most popular research areas in remote-sensing image processing recently. In particular, with the emergence of 5G and other new technologies, the capability of applications based on semantic segmentation of HRRSI has become stronger and more viable, such as land attribute classification [1], capacity identification and road extraction [2]. Various studies have been carried out, including multi-scale context learning [3,4,5,6,7,8,9,10], high-resolution restoration [11,12,13,14,15], and contextual relationships aggregation [16,17,18,19,20,21,22,23,24,25,26,27,28], and so on.

Previous work has demonstrated the importance of contextual information for semantic segmentation. Zhao et al. [3] and Chen et al. [4] extracted local contextual information at multiple scales by using parallel convolution or pooling operations with different sizes of receptive field. Zhao et al. collected global information at various scales under four different levels of pooling of operation, such as PPM [3]. Chen et al. considered it by using various atrous convolutions of different atrous rates. This was a classic work in this field that is still widely used nowadays. However, the network with a fixed structure was limited by the size of the local receptive field, so it could not effectively learn the long-range correlation. In order to overcome this constraint, Zhang et al. [16] proposed a context network based on an attention mechanism [29] which is used selectively to enhances the global contextual information. In addition, DA-Net [17], OC-Net [18], PSA-Net [20], CC-Net [21], ANN [23], ATTENTION_U-Net [11], GC-Net [24], CF-Net [25], SA-Net [30] and LA-Net [27] introduced contextual relations from various perspectives, respectively. Among these networks, ANN and OC-Net successfully reduced the computational complexity of an attention network without reducing the performance by simplifying the expressions of non-local operations. All these networks operated at pixel scale.

These pixel-level context models have had some success, but the overly detailed learning mode (pixel-by-pixel learning) can make the model sensitive [22]. Specifically, a large number of confusing features and unavoidable mislabeling can lead to confusion in the learning of contextual relations, which become the bottleneck of the pixel-based networks. Therefore, how to solve the problems induced by the pixel-level learning mechanism without degrading performance is the key to breaking through the bottleneck of the current semantic segmentation methods.

In recent work, ACFN-et [22], OCR-Net [19] and HMA-Net [26] modified the original pixel-level attention mechanism to the pixel-region/object ones and the performance of the network using the pixel-region attention mechanism has been improved. Specifically, they no longer calculate the correlation between each pixel, but calculate the correlation between each pixel and the corresponding region [26] or object [19] instead. These methods improved performance while reducing the amount of calculation. However, the relationship between pixels and regions is not enough to completely solve the confusion and the model is still relatively sensitive to some very similar features.

Our method constructs a region-enhanced semantic segmentation network for HRRSI, which makes the model focus on learning the region-level context instead of pixel-level context. Two modules, the region-enhancing module and the multi-scale context learning module, are carefully designed in the proposed network to realize the region-level attention mechanism. The region-enhancing module is composed of the region-correcting procedure and the regional context learning procedure. Regional context learning procedure is designed to learn the contextual relations at the regional level. The region-correcting procedure is designed to enhance the regional features by getting rid of the random pixels within a region and enhancing the heterogeneity between regions at the same time. Considering that the size of regions varies greatly in different images, especially in HRRSI, we designed the multi-scale context-learning module, ADAPTIVE_ASPP, to incorporate multi-scale information according to the size of regions to take advantage of the powerful learning ability of the ASPP module [4,6].

In addition, it should be noted that the multi-scale attention structure in the proposed method is different from scale perception [31], and the “pyramid multi-scale attention network” [5] recently released by NVIDIA, which belongs to post-processing and needs to copy the original structure when training the model. However, our method incorporates multi-scale information during model training, which does not require such complex and memory-consuming operations. 

We evaluated our approach on the Vaihingen and Postdam datasets of ISPRS, which are very challenging public datasets. In these experiments, the proposed method is superior to current popular methods, such as DEEPLABV3+ [6], DA-Net [17], OC-Net, PSP-Net [3], and most recently HMA-Net [26], LA-Net [27]. These segmentation methods for very high spatial resolution remote-sensing imagery have been widely used for land cover classification, building detection, and so on. 

The paper includes five parts. The first section is the introduction. Section 2 briefly introduces the related work. Section 3 elaborates our proposed model. Section 4 describes and analyses the experiments on three different public datasets, such as Vaihingen and Postdam. Section 5 draws the conclusion. 

## 2. Related Work

Multi-scale structure: PSP-Net and Deep-lab series [4,6] are the two classic multi-scale structures in the field of semantic segmentation. The former uses adaptive pooling with different pooling sizes, while the latter uses different atrous convolution to capture information at multiple scales. Sub-follow-up researchers continued to expand on these two structures and derived Dense-ASPP [32] with a denser scale, which, to some extent, solved the gridding problem caused by atrous convolution. At the same time, APC-Net [7] considered contextual relations at various scales by using PSP-Net structure. MFRN [8] uses a simplified version of the ASPP architecture to design a semantically segmented backbone network. In addition, the encoder structure similar to U-Net can also extract the multi-scale information of the network to a certain extent, such as V3+, AC-Net [9] and so on. However, the performance only using multi-scale structure is not satisfactory.

Contextual aggregation: the earlier Enc-Net [16] as Non-local [29]’s following work borrowed the attention mechanism to perform a weighted summation by balancing the importance of features. Subsequent studies learn contextual relations from a broader perspective. PSA-Net introduces the collecting and distributing branches to associate each pixel with the whole image to learn the pixel-level context. DA-Net further added a channel attention module to construct two parallel attention modules respectively at pixel-region levels and achieve better performance. By considering the usefulness of the pixel’s context, CC-Net designed a criss-cross attention module to learn the weight of the backbone position in the image instead of learning the weight of all position pixels. However, because the context is still learned at the pixel level, the complexity and performance of the model are not satisfactory. OCR-Net, ACF-Net and HMA-Net reduced the time complexity of the model and improved the segmentation accuracy by considering the contextual relationship between pixel and region. However, the trade-off between model complexity and accuracy is still an issue that needs to be carefully considered.

Boundary processing: GSCNN enhances the boundary prediction of the whole model by introducing a gating mechanism to perform the boundary prediction independently. DPC [10] applied boundary detection to improve the segmentation accuracy [12,33], which forces the improvement of boundary accuracy by adding extra tasks. Super-pixel semantic segmentation [28] attached heavier loss weight to boundary pixels, thus guiding the model. Dense-CRF [13] and Seg-Fix [34] improves the boundary by employing post-processing. All these methods require additional consumption. 

Coarse-to-fine scheme: many coarse-to-fine segmentation schemes have been developed [14,35,36,37,38]; among them, the classic one is to perform coarse segmentation on the intermediate features in the network [37], and then use the obtained segmentation result as a new feature representation to help the original network obtained more refined results.

Our method is similar to this kind of coarse-to-fine, which does not use coarse predictions as additional features or refine features, but uses it to learn the regional context information. 

Semantic segmentation of remote-sensing images: in the field of semantic segmentation of remote-sensing images, deep learning methods are quickly combined with traditional machine learning methods after the rapid development of deep learning methods. For example, TREE-U-Net [39] combines U-Net with decision tree structure. Super-pixel segmentation [28] realizes the combination of Deeplabv3+ and random forest. Although these methods have achieved good results, they have added additional modeling [40] and SCS-Net [41] has designed two different network structures without extra expense. In particular, due to the particularity of remote-sensing images, many remote-sensing images have more than three channels. Therefore, deep learning methods based on the information of the fourth channel or other channels are also derived, such as EEDFN [42]. HMA-Net and LA-Net combined the above two methods on the basis of pure deep learning and incorporating the additional feature information of a remote-sensing image. In addition, some researchers tried to add the boundary detection as a separate task. These methods either add additional calculations or require additional information.

Subsequently, our work is mainly focused on building a simpler and more efficient network without using additional information from remote-sensing images. Our network is similar to HMA-Net, which improves the performance of semantic segmentation of remote-sensing images by building a mixed multiple attention network. However, unlike HMA-Net, which focuses on contextual relationships between pixels and region, our network focuses on improving the integrity of the region and only use the three channels of the image as our input.

## 3. Method

**Figure 1:**
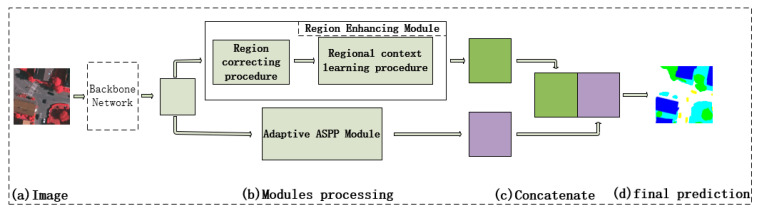
The proposed architecture of region-enhancing network (RE-Net). The upper branch is the region enhancing module, and the lower branch is the adaptive ASPP module. (**a**) the original image; (**b**) the modules processing process; (**c**) concatenate two features from corresponding module. (**d**) the final prediction of our work.

In order to further reduce the misclassification induced by the pixel-based method, we propose a region-enhancing network by taking the contextual relation information at regional scale instead of pixel scale. As shown in Figure 1, the architecture of the proposed network is mainly composed of region-enhancing module (REM) and the adaptive ASPP module. The RCM can be divided into the regional context learning procedure (RCLP) and the region correcting procedure (RCP) separately. The two procedures form the upper branch of the network by cascading and embedding. The RCLP is specially used for the network to learn the contextual relationship from the perspective of the region, while the RCP aims to enhance the consistency of the extracted features of each region, thus promoting the learning of the region-level context. In addition, the adaptive ASPP module takes the multi-scale information into consideration by automatically adjusting the weight of each scale information according to the size of initial retrieved region. Specifically, given a remote-sensing image, we first input it into the backbone to obtain the preliminary rough feature mapping, which is adjusted to be 1/8 of the origin image, the same setting as Chen et al. [4]. Then we input it into the region-enhancing module and the adaptive ASPP module, respectively. Finally, the outputs from the two modules are stacked as the inputs of the classifier for final segmentation.

### 3.1. Region-Enhancing Module

RCM is the primary structure used to enhance the regional feature instead of pixel information. RCM is composed of two procedures, RCLP and RCP. It should be noted that another RCP is embedded in the RCLP.

#### 3.1.1. Regional Context Learning Procedure

**Figure 2:**
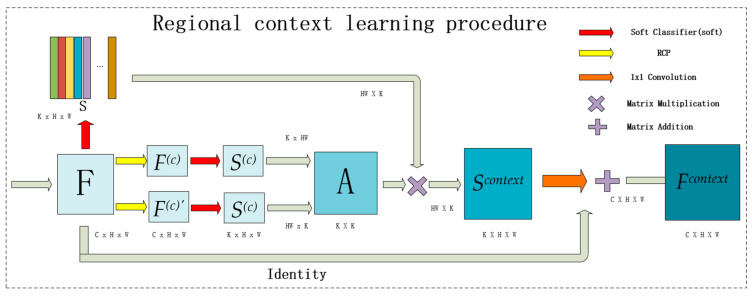
The details of proposed regional context procedure.

The purpose of the RCLP is to learn the region-level context and the flowchart of the procedure is shown in Figure 2. Specifically, the feature *F* without reginal context information extracted from the backbone network is processed by the RCLP and the feature *F^context^* learned regional context is subsequently retrieved. The detailed operations are as follows.

At first, the RCLP is inherited from the treatment of object characteristics in papers like OCR-Net [19] and ACF-Net [22]. These methods introduce the semantic segmentation procedure at both the middle and the last of its network to better extract the rough region-level features. Assume that the input features are *F* from the backbone network, which is pixel-level features, and *S* is the region-level features obtained after a soft classification of *F*. Equations (1) and (2) show the calculation of *S*.

(1) S=soft(F)

(2) $$S=\left\{s_{0},s_{1},\ldots ,s_{k-1}\right\},\left(i\in \left(0,k-1\right)\right)$$

where *s_i_* is the feature of the *i_th_* region, *k* represents the number of region, *soft* represents one 1 × 1 convolutional layer, the rough classifier.

The *RCP*, which greatly reduces the misclassified pixels to further improve the wholeness of region classification, is embedded explicitly into the RCLP to promote the learning of region-level context, and the RCP is described in detail at Section 3.1.2. We use $S^{\left(c\right)}$ to represent the region features after the RCP and rough classification, its calculation formula is shown in Equation (3).

(3) $$S^{\left(c\right)}=soft\left(RCP\left(F\right)\right)$$

Then, according to the non-local [29] method of context learning, the relationship matrix *A* between regions can be calculated as Equation (5).

(4) $$Z=reshape\left(S^{\left(c\right)}\right)$$

(5) $$a_{ij}=\sum_{d=0}^{HW}Z_{id}*Z^{T}{}_{dj},\;\;\left(i,j\epsilon \left(0,K\right)\right)$$

where $a_{ij}$ is the relationship between region *i* and *j*, *HW* represents the height and width of $S^{\left(c\right)}$ respectively. *reshape* represents the operation reshape $S^{\left(c\right)}$ from *K* × *H* × *W* to *K* × *HW*.

By connecting *S* with *A*, the region-level contexts are learned subsequently and the corresponding formula of the features (*S^context^*) after the regional context learning is defined as Equation (6).

(6) $$S^{context}=reshape\left({\left(reshape{\left(S\right)}^{T}*A\right)}^{T}\right)$$

where, *reshape* represents the operation rearranging the dimensions of the matrix to match the matrix multiplication. Specifically, reshape *S* from *K* × *H* × *W* to *K* × *HW*.

Finally, the obtained feature map $F^{context}$ with region-level context is restored to the dimension of the original feature map through a convolution, and an *Identity* is also added to avoid the ineffectiveness of the procedure according to the design of the residual module, which is defined as Equation (7).

(7) $$F^{context}=1\times 1\_conv\_up\left(S^{context}\right)+Identity$$

where 1 × 1*_conv_up* is the 1 × 1 convolution used to increase the number of channels. 

#### 3.1.2. Region Correcting Procedure

**Figure 3:**
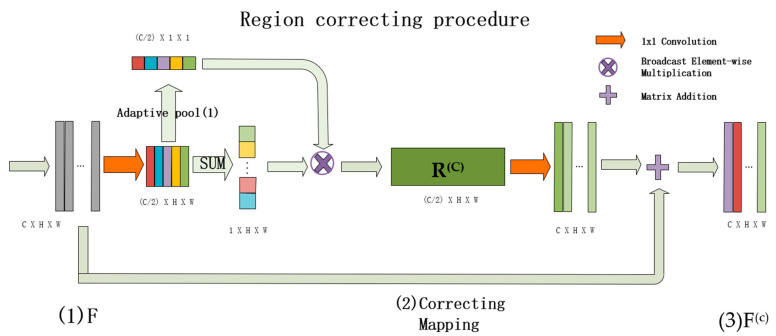
Detailed design drawing of the area correction procedure. Where, *adaptive pool(1)* represents the adaptive pooling operation of size 1. *SUM* represents the sum operation in channel dimension.

The overall structure of the RCP is shown in Figure 3. Firstly, each pixel’s features from the original feature map are accumulated to enlarge the differences of pixels belonging to different region and subsequently to remap the original image to obtain the corrected feature map *R^(c)^*, which can greatly improve the wholeness of the classified regions. An identity is added to *R^(c)^* to obtain the final corrected feature map *F^(c)^*. Specifically, *F* represents the original feature map. In order to reduce the computation loading, the number of channels is also reduced (1x1_conv_red)*,* so the calculation formula of *R^(c)^* is defined as Equation (8).

(8) $$R^{\left(c\right)}=\left(SUM\left(1\times 1\_conv\_red\left(F\right)\right)\right)⊗\left(adaptive\_pool\left(F\right)\right)$$

where 1 × 1_conv_red is the 1 × 1 convolution used to halve the number of channels. *Sum* represents the operation sum in the channel dimension. adaptive_pool represents an adaptive average pooling with size of 1.

The final feature map *F^(c)^* is calculated as Equation (9).

(9) $$F^{\left(c\right)}=F+1\times 1\_conv\_up\left(R^{\left(C\right)}\right))$$

### 3.2. Intra-Network Adaptive ASPP Module

The ASPP module is a classic multi-scale module that has been widely used for dealing with the multi-scale issues in the field of semantic segmentation. In this study, a simple change has been made to the ASPP module, which improve the efficiency of the network while dealing with the information extracted from the ASPP module. Compared to the original ASPP without considering the weight of each scale, the adaptive ASPP proposed in this study designs scale-based weight, which can be calculated by passing a region map classified from the initial feature map into two full connection layers.

### 3.3. Loss Function

In order to better compare with other studies, the simple and stable cross entropy loss function is chosen in this study, whose calculation formula is defined as Equation (10):

(10) $$CE\left(p,q\right)=-\sum_{i=1}^{C}p_{i}\log\left(q_{i}\right)$$

where *p_i_* represents the ratio of class *i* to the total sample, *q_i_* represents the probability that class *i* is predicted as class *i*, and *C* represents the number of categories.

At the same time, since we designed several structures, we added two auxiliary loss in the backbone and the REM in the network, respectively, to promote the learning of the network. The first auxiliary loss is added to the backbone after RES422B and it is composed of two 1 × 1 and one 3 × 3 convolution. The second auxiliary is added to the REM and it is composed of the simplest 1 × 1 convolution.

## 4. The Experiment

To verify our proposed model, we conducted a large number of experiments on four different open datasets, Vaihingen, Postdam, DLRSD, and GID. Among these datasets, Vaihingen and Postdam with only five classes have been released and well-evaluated for a long time; furthermore, the results using our model on the two datasets are compared with the results based on some other modes. Subsequently, the dataset with much more images and more classes (over 15 classes) including DLRSD and GID are also used for verification purpose. In this section, we will first describe the datasets used for verification purpose and their corresponding processing details. Secondly, we show our implementation in detail. Thirdly, some small experiments have been carried out to verify the effectiveness of the key procedures proposed in our network. Finally, our results on these two datasets are compared with the results based on the state-of-the-art methods.

### 4.1. Datasets

Vaihingen [43]: the dataset is composed of 33 orthorectified image tiles acquired over the town of Vaihingen (Germany), with an average size of 2494*2064 and a spatial resolution of 9 cm. Among the 33 images, 16 are fully annotated and distributed to the public, while the remaining ones comprise the test set and their ground truth is not released. The Vaihingen dataset contains five foreground classes and one background class. The five foreground classes include impervious surfaces (roads, concrete flat surfaces), buildings, low vegetation, trees, and cars. The background class is a class of “clutter” to group uncategorized surfaces and noisy structures. Classes are highly imbalanced, with the classes “buildings” and “impervious surfaces” accounting for roughly 50% of the data, while classes such as “car” and “clutter” account only for 2% of the total labels. In order to make a close comparison with other methods, we follow the previous work and mainly limit the research scope to the foreground scope. Meanwhile, in accordance with the setting on the official website of the ISPRS dataset, 16 of the datasets defined by the benchmark organizer were used as our training set and verification set, while the other 17 were used to test our model. It should be noted that we did not use the DSM data in this study.

Postdam [44]: the Potsdam dataset has the same foreground and background classes as the Vaihingen dataset. However, the Postdam dataset not only provides DSM data, but also provides more band combinations including RGB: 3 channels (R-G-B), and RGBIR: 4 channels (R-G-B-IR). In our experiment, the RGB combination is used in order to better compare with other research. The Postdam dataset contains a total of 38 high-resolution 6000*6000 remote-sensing images. According to the benchmark organization, 24 of them are used for training and verification purposes and 14 of them for testing purpose.

It needs to be pointed out that Vaihingen is a relatively small village and Potsdam a typical historic city with large building blocks, narrow streets and dense settlement structure; there are big differences between these two datasets.

DLRSD [45]: DLRSD is a dense labeling dataset that can be used for multi-label tasks, such as remote-sensing image retrieval and classification, and other pixel-based tasks like semantic segmentation. DLRSD has a total of 17 broad categories [45] with 100 images per class (1700 images totally), which is the same as the UC Merced archive, including aircraft, docks, jungle courts, buildings and so on. Among the 17 classes, the dock and airplane accounted for no more than 0.65% of the total data, which poses a great challenge to semantic segmentation. In this study, they are divided into training sets and verification sets by 4:1. The detailed information of DLRSD can be referred to [45].

GID: GID is a large-scale land-cover dataset constructed with Gaofen-2 (GF-2) satellite images, which has superiorities over the existing land-cover dataset because of its large coverage, wide distribution, and high spatial resolution. GID consists of two parts: a large-scale classification set and a fine land-cover classification set. In this study, the large-scale classification set containing 150 GF-2 images annotated at pixel-level is used for verification. The training and validation data with 15 categories is collected and they include farmland, meadow, lake, arbor woodland, paddy field and so on. Among them, the most challenging is shrub land, only accounts for 0.29% of the total dataset. GID and its reference annotations can be found online at GID Dataset (x-ytong.github.io) (accessed on 20 November 2019).

We will do major experiments on the Vaihingen and Postdam datasets, and then test the robustness of our model in GID and DLRSD, which owns more categories and more complicated scenarios.

It is important to note that we have not fine-tuned the model when we tested it on datasets other than Vaihingen.

### 4.2. Evaluation Indicators

In order to make a wider comparison with various research results, in addition to adopting the F1 score and overall accuracy (OA) used by the benchmark organization, we also took the mean intersection over union (MIoU) into account, which is one of the most used indicators in the field of semantic segmentation. Their definitions are at Equations (11)–(13) respectively.

(11) $$F_{1}=\left(1*\beta^{2}\right)*\frac{precision*recall}{\beta^{2}*precision+recall}$$

where β is used to balance recall and precision. In our experiment it was set to 1.

(12) $$MIoU=\frac{1}{k+1}\sum_{i=0}^{k}\frac{p_{ii}}{\sum_{j=0}^{k}p_{ij}+\sum_{j=0}^{k}pji-pii}$$

where *P_i_*_j_ represents the probability that class *i* is predicted to be class *j*, and *K* + 1 is the number of classes (including empty classes).

(13) $$OA=\frac{\sum_{i=0}^{k}P_{ii}}{\sum_{i=0}^{k}\sum_{j=0}^{k}p_{ij}}$$

Notably, similar to previous work, all of our evaluation indicators are calculated on the eroded data defined by the benchmark organization setters, in order to reduce the impact of uncertain border definitions on the evaluation.

### 4.3. Implementation Details

This study, ResNet50 is used as the backbone of the proposed network for better comparisons with the other methods because of its extensive use in sematic segmentation. The pretraining parameters’ settings of the ResNet50 are the same as the official training of PYTORCH. It should be noted that the setting of the backbone network is only for better comparison purposes with other studies. The backbone network can use any network, such as HR-NET [46,47]. In terms of learning rate, the momentum is set to 0.9, the weight attenuation is set to 4E-5, and the initial learning rate was set to 0.02. Synchronous batch-norm instead of the normal batch-norm is used in this study for taking distributed training for accelerating purpose. In terms of data enhancement, all the training images were randomly flipped horizontally or vertically, randomly scaled, and randomly cropped while conventional normalization and color dithering are applied to all the images in the meantime. All the images are cropped to 512*512. DeepLabv1 is used as the baseline network in this study.

We completed all our experiments on two 12GB NVIDIA TESLA P100.

### 4.4. Experiments on the Vaihingen Dataset

For the Vaihingen dataset, we clipped it by sliding window. The sliding window size was set to 512*512, the moving step size was set to 384 and the images smaller than 128*128 pixels were abandoned; 500 images were obtained. After five random scaling and clipping (0.5, 0.75, 1.25, 1.5, 2.0), 3 roasts (90,180,270), 1 random Gaussian blur, and 1 Gaussian noise, a data set of nearly 6500 images was obtained.

- Ablation experiments on multiple loss functions

**Figure 4:**
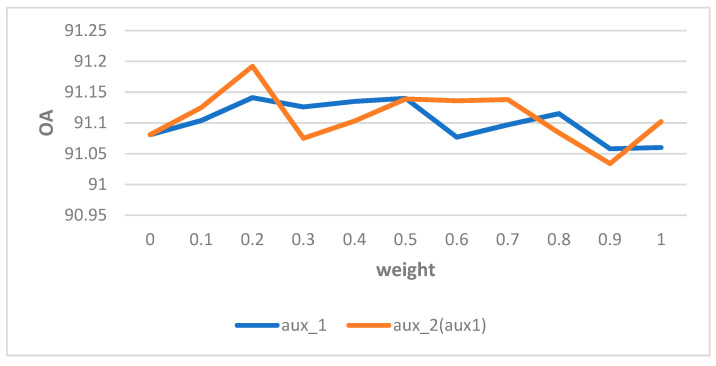
Comparison of different weight parameters. It is important to note that the experiment with AUX2 was based on the optimal weighting of AUX1 that had been selected.

Similar to previous studies, such as PSP-NET, the multiple loss functions were tested to facilitate training of the model. In detail, we added a DECODER after RES422B as auxiliary loss 1 (aux_1). In addition, a new loss function as auxiliary loss 2 (aux_2) was added after the region enhancing module. Taking the final CLS loss (main loss) counted, there were three losses in total. By fixing the proportion of the main loss function as 1.0, the OAs could be retrieved by changing the weights of aux_1 and aux_2 respectively from 0.1 to 1.0 with 0.1 interval. The results of the ablation experiments are shown in Figure 4.

Based on the results in Figure 4, the optimal weights for the two auxiliary loss functions were both 0.2. Based on the optimal weight settings of the auxiliary loss functions, the OA reached 91.192%, the MIOU reached 83.095%, and F1 score reached 90.616%, which were obvious improvements. We show the results of the ablation experiment in Table 1.

- Ablation experiments of various regional structures

In order to demonstrate the effectiveness of the proposed structures in the proposed method, ablation experiments for various regional structures were designed and implanted. Table 2 shows whether a simply designed self-adaptive ASPP module or a well-designed region enhancing module has achieved an obvious improvement compared with the baseline network. When only the adaptive ASPP structure is employed, the network is improved by 6.189 (MIoU), 2.223 (OA), and 3.996 (F1) respectively; meanwhile, the regional context learning module was applied alone, the improvements were 6.374, 2.302, and 4.546 for MIoU, OA, and F1 respectively; furthermore, the indicators were improved by about an extra 0.1, respectively, while combining the RCP and the RCLP together. When all the structures were combined, the accuracy of the network achieved the highest with MIoU of 83.095, OA of 91.192, and F1 score of 90.616. Compared with the baseline, the overall improvements were 7.086 (MIoU), 2.742 (OA), and 4.546 (F1) respectively.

- Comparisons with other contextual methods

In order to further demonstrate the effectiveness of our proposed method, the results on the Vaihingen dataset from the proposed method were compared with the results from some widely used methods by using contextual information, such as DeepLabv3+, PSP-Net, OCR-Net, and so on. As shown in Table 3, our method performs the best for all the three indicators. In the perspective of per-class, our method is the best on the two categories of building and car. It should be noted that our results are the only ones without any post-processing compared to the others.

As shown in Table 3, compared to other commonly used networks, our model is effective in the segmentation of all categories and the per-class F1 score for each category is very close or the best; therefore, the OA, MIoU, and F1 index are all the highest ones. Especially, the smallest object, car, reaches the highest accuracy. However, it has to be noted that the low vegetation and trees are the least classified categories because of their high similarity under RGB color mode.

- Complexity comparisons with other similar modules

We compared the efficiency of our model with the efficiencies of the other similar context models. We measured the increased parameters that were introduced by the context modules, shown in Table 4. Most relational context schemes require less parameters compared with the multi-scale context schemes. For example, our REM(OA of 91.20) only requires 1/5 of the parameters of PPM(OA of 88.99) and 1/4 of the ASPP(OA of 88.91). Meanwhile, although the amount of parameter of PAM(included in DA-Net) is less than that of ours, its accuracy, OA of 88.59 is lower than ours [17]. Thus, it shows that the efficiency of the REM is quite good. In general, our REM is a much better choice if we consider the balance between performance and memory complexity.

- Visualize results

**Figure 5:**
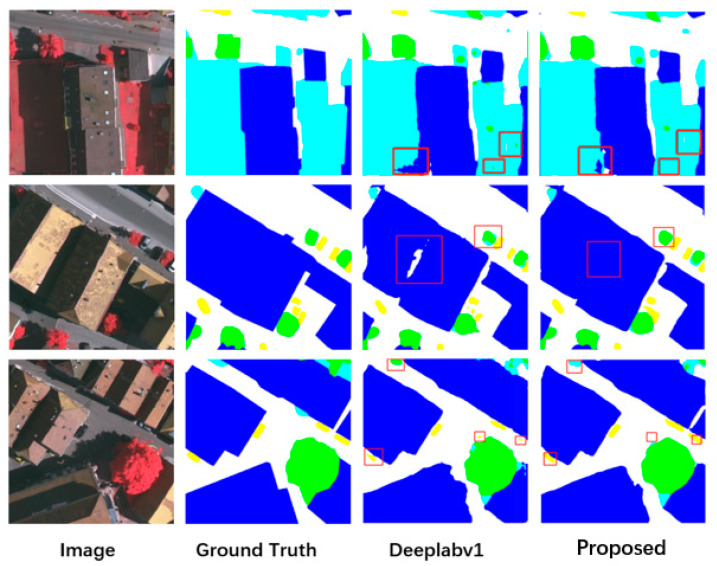
Examples of semantic segmentation results from the Vaihingen. (ground truth, the label published by the publisher of the open dataset).

We display part of the results of the baseline network (DeepLabv1) and our RE-Net in Figure 5 and this shows that our results are clearly better than those from the baseline network in different cases, especially in the red boxes. Since the region enhancing module has considered the pixels in a whole, the regional integrity of our results is much better than that from the baseline network. At the same time, in the recognition of smaller objects, such as car, the region enhancing network is also better. However, the pixels at the boundary of the region are still difficult to predict accurately. When there are complex boundaries in the image, as in other people’s work, we still cannot obtain very good results.

### 4.5. Experimental Results on Postdam Dataset

Some similar work [19,22] has already yielded excellent results on different datasets, such as Cityscapes [19,22], ADE20K [19] and COCO-Stuff [19]. In order to further test our model’s robustness, without any modifications, we conducted similar experiments on the Postdam dataset of ISPRS and compared the results with those from the current popular methods. As shown in Table 5, our model achieves the best performance on MIoU and OA. The LA-Net has the highest F1 score and the F1 score of our results is very close to that of the LA-Net. The values of the three indicators (MIoU, OA and F1 score) from our results are 84.93%, 91.37%, and 91.66%, respectively, based on the test on the Postdam dataset.

Based on the test of the Postdam dataset, the segmentation of cars is still as good as that with the Vaihingen dataset, which indicates that our model performs very accurately in segmenting small targets. However, the confusion between low vegetation and trees is still one of the limitations of our model because the difference between low vegetation and trees within the remote sensing imagery with only RGB bands is very small. Since trees are much higher than low vegetation, the shadow of trees can be a good indicator to be used for separating the two categories and it will be used in our next study. In addition, categories with complex and unsharp boundaries are still bottlenecks, which limit the performance of our model.

### 4.6. Experimental Results on the Other Datasets

We conducted further tests on two datasets without any fine-tuning. These datasets are more complicated than Postdam and Vaihingen, which contain more images and have many more classes.

**Figure 6:**
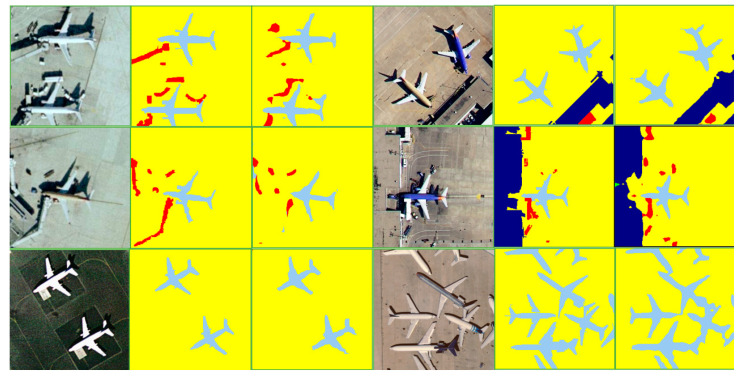
Examples of semantic segmentation results from the DLRSD, (from left to right are: the original image, the ground truth, and the predicted images).

**Figure 7:**
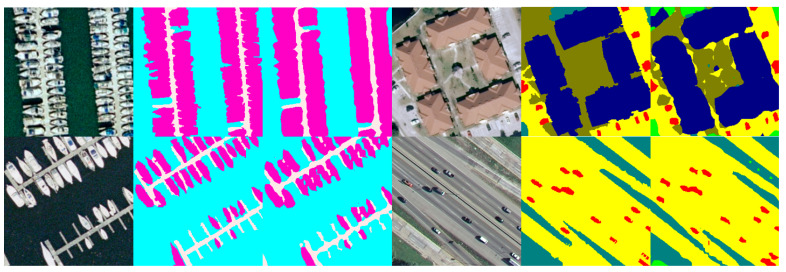
Examples of semantic segmentation results from the DLRSD, (from left to right are: the original image, the ground truth, and the predicted image).

Table 6 shows the results of RE-Net on DLRSD. Figure 6 and Figure 7 show the comparison between the predicted classes from our model and the annotated classes from the dataset, and the most challenging classes including airplane and dock are presented, which only accounts for 0.65% of the total annotations. RE-Net has also achieved very good results other datasets with much different categories, such as court, field, pavement, ship, thanks, and water. An overall accuracy of about 90% has been achieved for these categories. In particular, the accuracy of field and sea both reaches an astonishing accuracy of 99.5%. Although the number of classes reach 17 and they are tri-folds of Postdam and Vaihingen, the overall accuracy reaches 86.04, which proves that our model is still efficient when dealing with different resolution images, completely different categories and scenes.

Although the number of classes in the four datasets are very different, some of the classes are exactly the same or very similar; therefore, we would like to compare the segmentation accuracy of these classes. The Vaihingen, Postdam, and DLRSD all have three categories that are the same including cars, trees, and buildings. The Vaihingen and Postdam datasets achieved very similar per-class F1 scores for each of these classes because they have the same spatial resolution. The per-class F1 scores for cars, trees, and buildings are (90.01, 89.42, and 96.24) and (96.11, 85.94, and 96.3) on the Vaihingen and Postdam datasets, respectively, and the difference may be caused by the labeling and numbers of samples. Although the DLRSD has relatively lower spatial resolution, the per-class F1 scores have reached 83.5, 86.72, and 88.94 for cars, trees, and buildings, respectively. Among the three categories, the cars’ accuracy is lowered the most because it is the smallest object within all these categories. This also reminds us that some of the classes from different datasets can be incorporated into samples for comprehensive training to further improve the model’s applicability and accuracy.

At the same time, our model was used on GID for land-cover classification and the results are presented in Table 7. The overall accuracy reached 91.27, the F1 score reached 88.12, and the MIOU reached 79.38. Shrub land reached an accuracy of 78.45, whose data volume only accounts for 0.25%. The experiment also verified the effectiveness of our method well.

RE-Net has achieved almost the highest accuracy on the Vaihingen dataset for all the indicators; the OA, F1 index and MIoU are 91.20, 90.62, and 83.10, respectively. Compared with the baseline, the accuracy has been improved by 7.086, 2.742, 4.546 for OA, F1 index, and MIoU, respectively. At the same time, it was tested directly on the other three public datasets without any fine-tuning and they all obtained very good results. The three indicators (OA, F1 index, and MIoU) are (91.37, 91.66, and 84.93), (86.04, 85.99, and 76.18), and (91.27, 88.12, and 79.38) for the Postdam, DLRSD, and GID, respectively. In addition, it should be noted that, compared with other common structures (like PPM, ASPP, etc.), the RE-Net only uses 0.8043M parameters which is much less than the 3.3495M of ASPP, the 4.1984M of PPM and so on.

## 5. Conclusions

Deep learning models have shown their advantages on semantic segmentation for remote-sensing image and the early models based on pixel-level context have achieved some success; however, the overly detailed learning mode (pixel-by-pixel learning) can make the model sensitive. Specifically, a large number of confusing features and unavoidable mislabeling can lead to confusion on the learning of contextual relations, which become the bottleneck of the pixel-based networks. Therefore, in order to solve the problems induced by the pixel-level learning mechanism without degrading performance, a new model for enhancing regional integrity is proposed in this study. The region-enhanced network starts from the contextual relationship between regions, so the network mainly learns the contextual relationship between regions as much as possible, which does improve the accuracy of the segmentation. A large number of experiments of our network on the Vaihingen and Postdam benchmark datasets from ISPRS show that our approach brings consistent improvements. Furthermore, the dataset with many more images and more classes (over 15 classes) including DLRSD and GID are also used for testing and the results demonstrate that the proposed model is also applicable to the complicated datasets without any fine-tuning. Our contribution can be summarized as follows:

(1) We designed a simple region-enhancing module to enhance the integrity of each region; specifically, the embedded regional context learning procedure learns the context relationship at the regional level. Meanwhile, the region correcting procedure explicitly corrects the features of separated pixels by operating aggregation based on the initial and rough features extracted from the backbone, which enhances both the consistency within the region and the differences between regions and facilitates the work of regional context learning.
(2) The in-network adaptive ASPP module is designed to focus on the multi-scale problem of remote-sensing images by considering the scale-based weights. That is, when the area existing in the image is large, more attention will be paid to the features at a larger receptive field, which greatly improves the robustness of the model while not increasing complexity.

Based on the experiments on three different datasets, it can be concluded that our model is effective and efficient for remote-sensing imagery segmentation for most of the categories. However, for some categories, such as low vegetation and trees, which appear to have little difference in RGB style in most of the datasets, the proposed model still performs poorly. In order to improve the recognition rates, we found that the trees are always accompanied with shadows but low vegetation is not, which is induced by the height of the trees; therefore, the shadows can be used for better separating low vegetation and trees in our next study. Furthermore, the shadows from different categories are very similar, so the classification of shadows also influences the segmentation accuracy significantly; subsequently, our future studies will continue to focus on the segmentation of shadows.

## Figures and Tables

**Table 1:** Ablation study for multiple loss functions.

	Backbone	Aux_1	Aux_2	Main Loss	MIOU	OA	F1
RE-Net	Resnet50			✓	82.889	91.081	90.488
RE-Net	Resnet50	✓		✓	83.054	91.141	90.588
RE-Net	Resnet50	✓	✓	✓	83.095	91.192	90.616

**Table 2:** Ablation study for various regional structures.

Model	MIOU	OA	F1
Deeplabv1(baseline)	76.006	88.450	86.070
+adptive_aspp	82.189	90.673	90.066
+rcontext	82.380	90.752	90.182
+rcontext +rc	82.465	90.904	90.232
+all	83.095	91.192	90.616

**Table 3:** The results of each model on Vaihingen. MIOU, the mean intersection over Union; OA, the overall accuracy.

Method	Backbone	Per-Class F1 Score(%)					MIOU	OA	F1
		Impervious Surface	Building	Low Vegetation	Tree	Car			
Deeplabv3+	Resnet50	91.35	94.34	81.32	87.84	81.31	——	88.91	86.60
PSP-Net	Resnet50	91.44	94.38	81.52	87.91	78.02	——	88.99	86.65
OCR-Net	Resnet50	92.75	95.98	83.65	89.16	89.21	82.32	90.82	90.15
DA-Net [17]	Resnet50	90.78	94.11	81.40	87.42	75.85	——	88.59	85.91
ACF-Net	Resnet101	92.93	95.27	84.46	90.05	88.64	82.68	90.90	90.27
TreeUNet [39]	——	92.50	94.90	83.60	89.60	85.90	——	90.40	89.30
LA-Net [27]	Resnet50	92.41	94.90	82.89	88.92	81.31	——	89.83	88.09
HMA-Net [26]	VGG16	91.86	94.52	83.17	89.81	87.15	——	89.98	80.68
CC-Net	Resnet101	93.29	95.53	85.06	90.34	88.70	82.76	91.11	90.58
CASIA2 [41]	Resnet101	93.20	96.00	84.70	89.90	86.70	——	91.10	90.10
BKHN11	Resnet101	92.90	96.00	84.60	89.90	88.60	——	91.00	90.40
RE-Net	Resnet50	93.27	96.24	84.14	89.42	90.01	83.10	91.20	90.62

**Table 4:** Comparison with other similar work in terms of the number of parameters.

Method	ASPP	PPM [3]	SA [29]	PAM [17]	OCR	RCCA [21]	REM(Ours)
Params(M)	3.3495	4.1984	2.1022	0.3283	14.8357	2.1433	0.8043

**Table 5:** The results of each model on the Postdam dataset.

Method	Backbone	Per-Class F1 Score(%)					MIOU	OA	F1
		Impervious Surface	Building	Low Vegetation	Tree	Car			
Deeplabv3+	Resnet50	92.35	96.77	85.22	86.79	93.58	——	89.74	90.94
FCN	Resnet50	90.98	94.10	81.25	87.58	76.80	——	88.66	86.14
FCN+SE [48]	Resnet50	90.43	93.95	81.33	87.50	63.33	——	88.27	83.31
FCN+BAM [49]	Resnet50	90.77	94.01	81.54	87.78	71.76	——	88.62	85.17
FCN+CBAM [50]	Resnet50	90.86	94.03	81.16	87.63	76.26	——	88.64	85.99
FCN+GloRe [51]	Resnet50	90.57	93.99	81.28	87.49	70.09	——	88.41	84.68
PSP-Net	Resnet50	91.61	96.30	86.41	86.84	91.38	——	89.45	90.51
DA-Net	Resnet50	91.61	96.44	86.11	88.04	83.54	——	89.72	89.14
UZ_1 [48]	——	89.30	95.40	81.80	80.50	86.50	——	85.80	86.70
UFMG_4 [52]	——	90.80	95.60	84.40	84.30	92.40	——	87.90	89.50
LA-Net	Resnet50	93.05	97.19	87.30	88.04	94.19	——	90.84	91.95
RE-Net	Resnet50	93.33	96.30	86.63	85.94	96.11	84.93	91.37	91.66

**Table 6:** The results of RE-Net on DLRSD.

	Airplane	Bare-Soil	Build-ings	Cars	Chap-arral	Cour-t	Dock	Field	Grass	Mobile Home	Pavement	Sand	Sea	Ship	Than-ks	Trees	Water	
OA	88.65	73.61	88.94	83.5	70.38	92.55	73.56	99.58	80.81	78.88	90.22	82.28	99.59	91.46	94.18	86.72	92.1	86.04
F1	85.33	72.79	87.11	84.23	72.38	88.7	75.51	99.75	81.82	81.61	90.32	87.32	97.71	87.74	90.24	86.02	93.35	85.99
IOU	74.41	57.23	77.16	72.76	56.71	79.7	60.65	99.5	69.24	68.94	82.35	77.5	95.52	78.16	82.21	75.45	87.53	76.18

**Table 7:** The results of RE-Net on GID.

	Industrial Land	Urban Residential	Rural Residential	Traffic	Paddy	Irrigated	Dry Cropland	Garden Plot	Arbor Woodland	Shrub Land	Natural Grassland	Artificial Grassland	River	Lake	Pond	
OA	82.26	88.9	76.75	87.52	94.11	97.19	78.67	76.68	99.22	78.45	98.29	92.24	97.25	97.5	74.44	91.27
F1	83.12	87.98	79.77	88.72	90.07	95.76	84.14	81.04	99.36	81.28	98.28	85.36	97.55	86.89	82.46	88.12
IOU	71.11	78.54	66.35	79.72	81.94	91.87	72.62	68.12	98.72	68.46	96.63	74.46	95.22	76.83	70.16	79.38

## Data Availability

The data presented in this study all are openly available: Vaihingen at 2D Semantic Label.-Vaihingen (isprs.org, accessed on 25 October 2021), [43], Postdam at 2D Semantic Labeling-Potsdam (isprs.org, accessed on 25 October 2021), [44], DLRSD at https://sites.google.com/view/zhouwx/dataset#h.p_hQS2jYeaFpV0, accessed on 25 October 2021, [45] and GID at GID Dataset (x-ytong.github.io, accessed on 25 October 2021).
